# Cancer risks among BRCA1 and BRCA2 mutation carriers

**DOI:** 10.1038/sj.bjc.6603535

**Published:** 2007-01-09

**Authors:** E Levy-Lahad, E Friedman

**Affiliations:** 1 Institute of Medical Genetics, Shaare Zedek Medical Center, Hebrew University Medical School, Jerusalem, 91031, Israel; 2 Oncogenetics Unit, Chaim Sheba Medical Center, Tel-Hashomer 53621; Sackler Faculty of Medicine, Tel Aviv University, Tel Aviv, 69978, Israel

**Keywords:** BRCA1, BRCA2, penetrance, breast cancer, ovarian cancer

## Abstract

BRCA1 and BRCA2 mutations increase breast and ovarian cancer risks substantially enough to warrant risk reduction surgery, despite variable risk estimates. Underlying this variability are methodological issues, and also complex genetic and nongenetic effects. Although many modifying factors are unidentified, known factors can already be incorporated in individualised risk prediction.

BRCA1 and BRCA2 were identified as genes mutated in hereditary breast/ovarian cancer by genetic analysis in families with multiple cases of these malignancies. In the following decade, as BRCA1 and BRCA2 testing became more common in patients with a personal or family history of cancer, numerous studies assessed cancer risks in carriers of these mutations. These studies have resulted in variable risk estimates, leading to difficulties in defining risk figures that are clinically applicable to the individual patient. However, they illuminated the complexities that underlie risk prediction even in the context of a single gene with major effects on cancer predisposition. The emerging picture is that breast and ovarian cancer risks in BRCA1 and BRCA2 carriers are substantially higher than in the general population, but that they are considerably affected by nongenetic, environmental factors, and by additional genetic modifiers.

## BREAST AND OVARIAN CANCER RISK IN WOMEN

Original estimates of breast and ovarian cancer risks in carriers were based on the families used for positional cloning of the BRCA1 and BRCA2 genes, where all carriers had molecular testing, and all cancer diagnoses were validated. These families were chosen because they harboured multiple, early-onset cases, that is, they were essentially selected for high cancer risk. In the Breast Cancer Linkage Consortium (BCLC) families, by age 70 years, BRCA1 carriers had a breast cancer risk of 71% (95% CI 53–82%) and an ovarian cancer risk of 47–63% (Easton *et al*, 1995). BRCA2 carriers had a breast cancer risk of 84% (95% CI 43–95%), and an ovarian cancer risk of 27% (95% CI, 0–47%) (Ford *et al*, 1998). Many subsequent studies were case-based, that is, risk was estimated by analysis of family history in relatives of breast or ovarian cancer patients, often from hospital-based series (Antoniou *et al*, 2003). The design of these studies partially addressed the ascertainment bias inherent in studying highly selected, multiple-case families, but risk estimates were based on family history, rather than on testing carriers directly and validating their cancer status. With this design, misclassification can result from discrepancies between modelled *vs* true carrier status, and from errors in cancer reporting by relatives, a particularly common problem for ovarian cancer. In a meta-analysis of such case-based studies, by age 70 years, in BRCA1 carriers breast cancer risk was 65% (95% CI 51–75%) and ovarian cancer risk was 39% (95% CI 22–51%), and in BRCA2 carriers breast cancer risk was 45% (95% CI 33–54%) and ovarian cancer risk was 11% (95% CI 4.1–18%) (Antoniou *et al*, 2003). One study, performed in American Ashkenazi Jews (AJ), combined ascertainment of unselected breast cancer cases with direct genetic testing and clinical status validation in relatives (King *et al*, 2003). By age 70 years, for BRCA1 carriers, breast cancer risk was 69% (SE 5%) and ovarian cancer risk was 46% (SE 6%), and for BRCA2 carriers, breast cancer risk was 74% (SE 8%) and ovarian cancer risk was 12% (7%). These risks continue to rise at older ages, reaching ∼80% for breast cancer at age 80 years (Easton *et al*, 1995; Ford *et al*, 1998; King *et al*, 2003). Thus, point estimates of risk are somewhat lower in case-ascertained, rather than family-ascertained studies, but because of the large confidence intervals, substantial overlap exists and many differences are not statistically significant. However, if ‘true’ risk estimates are those for any carrier in the population, even case-ascertained studies may be biased, because the very occurrence of cancer in a carrier suggests that other breast/ovarian cancer risk factors will be over-represented in her relatives, leading to overestimation of risk in carriers (Begg, 2002). These biases can be fully addressed only by population-based studies, which currently are technically feasible only in ethnic groups with founder mutations, for example, AJ and Iceland, where a few, easily tested mutations account for practically all deleterious changes in BRCA1 and BRCA2. A study of 5318 AJ volunteers identified 120 carriers (93 cancer free), and estimated cancer risk by comparing their family history with that of non-carriers (Struewing *et al*, 1997). Cancer risks were similar in BRCA1 and BRCA2 carriers, and by age 70 years breast cancer risk was 56% (95% CI 40–73%), and ovarian cancer risk was 16% (95% CI 6–28%). While breast cancer risk is in range of other estimates, ovarian cancer risk is substantially lower, perhaps because of unverified family history. Also, cancer rates in non-carriers were relatively high in this study, which may have led to deflated risk estimates based on comparing carriers to non-carriers. Discussing all published studies of breast and ovarian cancer risk in BRCA1 and BRCA2 carriers is beyond the scope of this review, but we would like to address the variability of risk estimates in the literature. Some of the variation is clearly methodological: family-based studies are prone to ascertainment bias, and almost all case- and population-based studies performed so far could be prone to misclassification of genetic and clinical status. However, some of the variation is likely due to true biological causes, including effects of specific mutations (allelic effects), other genetic factors, and nongenetic, environmental influences. The assumption is that in sufficiently large studies these variations would be equally distributed and risk estimates would remain comparable, but sample sizes are not large in all studies, and some risk-affecting factors can differ significantly, especially when studies are performed in specific ethnic groups or in different countries.

## EFFECTS OF SPECIFIC MUTATIONS

BRCA1 and BRCA2 are large genes, with thousands of different mutations. A clear genotype–phenotype correlation exists for BRCA2 mutations, where the central part of the gene (nt 3035–6629 in exon 11) is named the ovarian cancer cluster region (OCCR) because mutations within it are associated with an increased ovarian: breast cancer ratio. Compared to other BRCA2 mutations, OCCR mutations are associated with higher ovarian cancer risk (RR=1.88; 95% CI 1.08–3.33) and lower breast cancer risk (RR=0.63; 95% CI 0.46–0.84) (Thompson and Easton, 2001). Similar studies on BRCA1 mutations are less conclusive, but suggest that mutations in the central part of the gene (nt 2401–4190) are associated with lower breast cancer risk (RR=0.71; 95% CI 0.58–0.86), and that mutations in the 3′ end (beyond nt 4191) could be associated with lower ovarian cancer risk (RR=0.81; 95% CI 0.66–1.00) (Thompson and Easton, 2002). Most studies have not taken these general genotype–phenotype correlations into account. Furthermore, specific mutations could have unique risk profiles, and their relative prevalence in different studies could affect final risk estimates. In this context, one should note the locations of the common AJ and Icelandic mutations, which constitute a substantial fraction of carriers in many studies. In BRCA1, the common Jewish 185delAG and 5382insC mutations are outside the low breast cancer risk central region, and 5382insC is within the low ovarian cancer risk region. In BRCA2, the common Jewish mutation, 6174delT, is within the OCCR, whereas the common Icelandic mutation, 999del5, is outside the OCCR.

## OTHER GENETIC FACTORS – GENETIC MODIFIERS

The phenotypic expression of inherited disease can vary widely even between individuals with the same disease-causing mutations, as a result of both nongenetic variation and polymorphisms/mutations in other genes, known as genetic modifiers. There is epidemiological evidence for the existence of such modifiers for BRCA1 and BRCA2 mutations, since there is familial clustering of the cancer site: carriers in families with ovarian cancer index cases are at higher risk for ovarian cancer and at lower risk for breast cancer than carriers from families with breast cancer index cases (Easton *et al*, 1995; Antoniou *et al*, 2003; Simchoni *et al*, 2006). In the case of multiple generation families with different environments, familial clustering is likely to represent sharing of genetic modifiers within families. The search for genetic modifiers was originally limited to candidate genes, especially genes related to hormone metabolism and pathways involving BRCA1 and BRCA2. Length of repeat variations in the androgen receptor gene and in the steroid nuclear receptor coactivator AIB1/NCOA3 were reported to affect breast cancer risk (reviewed in Narod, 2002a), but this has not been confirmed (Spurdle *et al*, 2005, 2006). A noncoding polymorphism in RAD51, which binds BRCA2 in the DNA repair process, has been shown to increase breast cancer risk in BRCA2 carriers (HR=3.2−5.5) in three independent studies, all performed in AJs (discussed in Simchoni *et al*, 2006). There has been one report of an HRAS-linked polymorphism affecting ovarian cancer risk in BRCA1 carriers, but no follow-up studies were published (Narod, 2002a). Thus, the only confirmed genetic modifier is RAD51 in AJ BRCA2 carriers. Whole genome analysis strategies are now being applied and may yield significant genetic modifiers. Because genetic modifiers are yet to be identified, they have not been accounted for in current risk-estimate studies.

## NON-GENETIC EFFECTS

There are very significant nongenetic effects on breast and ovarian cancer risk in BRCA1 and BRCA2 carriers. This is evidenced by the consistent observation of increasing breast and ovarian cancer risks in carriers born in more recent years (Antoniou *et al*, 2003; King *et al*, 2003), although they carry the same mutations (and genetic modifiers). For example, an RR of 7.7 (95% CI 2.6–23) for breast cancer risk was found in BRCA1 carriers born from 1960 on, compared to those born before 1920 (Antoniou *et al*, 2003). Further environmental influences are suggested by the effect of country of origin on cancer risk (Antoniou *et al*, 2003). Investigation of specific nongenetic factors has focused on factors known to affect risk for sporadic breast and ovarian cancer, for example, reproductive behaviour, hormonal exposure, and lifestyle habits (Narod, 2002a; Dumitrescu and Cotarla, 2005). Risk reduction surgeries (salpingo-oophorectomy and mastectomy) are commonly offered to carriers, and result in substantial, nongenetic decrease in breast/ovarian cancer risk. They are not discussed here, since most studies censor carriers at the time of first preventive surgery.

### Reproductive factors

#### Age at menarche

Younger age at menarche is associated with increased risk for sporadic breast cancer. An effect was not observed in BRCA2 carriers, but BRCA1 carriers whose age at menarche was 14–15 years had a 54% reduction in breast cancer risk compared to those with menarche at ⩽11 years of age (OR=0.46, 95% CI 0.30–0.69) (Kotsopoulos *et al*, 2005).

#### Pregnancy

Increased parity is protective for sporadic breast cancer beyond age 40 years, but may increase risk for early-onset breast cancer. In BRCA1 and BRCA2 carriers, parity effects may also be age dependent. While an early report suggested that parity may increase risk for early onset (<40 years) breast cancer in BRCA1 carriers (Narod, 2002a), a larger retrospective study of 1260 carrier pairs by the same group did not confirm this finding, and even observed decreased breast cancer risk in BRCA1 carriers with ⩾four children (OR=0.62, 95% CI 0.41–0.94, *vs* nulliparous carriers) (Cullinane *et al*, 2005). In BRCA2 carriers, this study found that parity caused a borderline increase in risk for breast cancer before age 50 years (OR=1.17 for each pregnancy, 95% CI 1.01–1.36) (Cullinane *et al*, 2005). In a case-only study, young age at first pregnancy delayed onset of breast cancer in carriers (King *et al*, 2003), and a retrospective study of 1601 carriers found that in women over 40 years of age, each full-term pregnancy reduced breast cancer risk by 14% (95% CI 6–22%). An age effect was seen in that BRCA2 carriers with later first pregnancies had increased risk, whereas BRCA1 carriers with first birth over age 30 years were at lower risk than those with first birth before age 20 years (Andrieu *et al*, 2006a). Overall, the effect of parity on breast cancer in carriers is similar to that in sporadic cases.

#### Breast feeding

In a case–control study of 965 BRCA1 and 280 BRCA2 pairs, breast feeding did not influence breast cancer risk in BRCA2 carriers, but BRCA1 carriers who breast fed for over 1 year were less likely to have had breast cancer than those who never breast fed (OR=0.55, 95% CI 0.38–0.80) (Jernstrom *et al*, 2004). The retrospective cohort study of 1601 carriers did not show any breast feeding effect (HR=0.89, 95% CI 0.62–1.27) (Andrieu *et al*, 2006a).

#### Hormonal exposures – oral contraceptives (OC)

The effect of OC on breast cancer risk in carriers is difficult to assess because of changing patterns of use and OC formulations with time. In a retrospective case–control study of 981 BRCA1 and 330 BRCA2 carrier pairs, there was no effect of OC on breast cancer risk in BRCA2 carriers, but in BRCA1 carriers risk for early-onset (<40 years) breast cancer was increased in women who first used OC before 1975 (OR=1.42, 95% CI 1.17–1.75), who used OC before age 30 years (OR=1.29, 95% CI 1.09–1.52), or who used them for 5 or more years (OR=1.33, 95% CI 1.11–1.60) (Narod *et al*, 2002b). Two recent studies based on early-onset breast cancer cases, including small numbers (<100) of carriers, found no general increase in breast cancer risk with OC use. One study found increased breast cancer risk only with teenage (<20 years) OC use (OR=1.53 (95% CI 1.17–1.99) per year of use at age <20 years) (Jernstrom *et al*, 2005), and in the others there was even a suggestion of decreased breast cancer risk within BRCA1 carriers (OR=0.22, 95% CI 0.1–0.49 for OC use of at least 1 year) (Milne *et al*, 2005). Altogether, these studies suggest that for breast cancer risk, OC have similar relative effects in carriers and non-carriers. These effects may be attenuated in newer, low-dose preparations, but the absolute effect may be higher in carriers. OC use is known to decrease ovarian cancer risk in the general population, and similar effects have been seen in most studies of carriers (Narod, 2002a). In a case–control study, ovarian cancer risk was reduced by 15% (OR=0.85, 95% CI 0.53–1.36) with at least 1 year of OC use, and by 38% with 6 years or more of OC use (OR=0.62, 95% CI 0.35–1.09) (Whittemore *et al*, 2004).

#### Physical activity and body weight

Most of the evidence suggests that in women at average risk, physical activity, and lack of obesity are protective against breast cancer. In a study of 48 unaffected carriers and 89 carriers with breast cancer, physical activity was not protective (Nkondjock *et al*, 2006), but caloric intake in the highest *vs* the lowest tertile increased breast cancer risk (OR 2.76, 95% CI 1.10–7.02). A larger case-only study found that increased physical activity and lack of obesity in adolescence were associated with significantly delayed breast cancer onset in BRCA1 and BRCA2 carriers (King *et al*, 2003).

#### Radiation exposure

Early radiation exposure increases breast cancer risk, raising the concern that early screening mammography in carriers may be harmful. In a case–control study of 3200 carriers, previous mammography did not increase breast cancer risk (OR 1.03, 95% CI 0.85–1.25) (Narod *et al*, 2006). In contrast, a retrospective cohort study of 1601 carriers found that any exposure to chest X-rays was associated with an increased breast cancer risk (HR=1.54; *P*=0.007). Risk was increased in carriers aged 40 years and younger (HR=1.97; *P*<0.001), particularly those exposed only before the age of 20 years (HR=4.64; *P*<0.001) (Andrieu *et al*, 2006b). Further studies may determine if these results imply differential effects of dose and developmental timing of radiation exposure.

While fewer factors seem to significantly influence BRCA2- compared to BRCA1-related risks, this could be the result of smaller sample sizes of BRCA2 carriers and subsequent lack of power. The demonstration of multiple nongenetic effects on cancer risk in carriers requires controlling for these factors in risk assessment. Year of birth is now commonly included in risk models, but is unlikely to account for all environmental effects.

## MALE-SPECIFIC CANCER RISKS – MALE BREAST AND PROSTATE CANCER

Data on male-specific cancer risks are much more limited than on female-specific cancer risks.

*Male breast cancer (MBC)* is a major characteristic of the BRCA2-associated cancer syndrome. Cumulative risk for this malignancy was assessed in 164 BRCA2-BCLC families, which included 59 cases of male breast cancer, and risk was estimated at 2.8% (95% CI 0.6–13.0%) by age 70 years, rising to 6.9% by age 80 years (95% CI 1.2–38.6%) (Thompson and Easton 2001). This corresponds to a RR of ∼80-fold. Mutation location (within or outside the OCCR) does not influence MBC risk (Thompson and Easton, 2001). While BRCA1 carriers are at lower risk than BRCA2 carriers, they seem to be at increased risk compared to the general population. Cumulative MBC risk in BRCA1 carriers has been estimated at 5.8% (95% CI 1.3–10.4%) over the entire lifetime (to age >80 years), a figure based on four cases among 102 male carriers in a clinic-based study (Brose *et al*, 2002). In studies of MBC cases, the rate of BRCA1 mutations has been 3.2–10.4% (reviewed by Liede *et al*, 2004) significantly higher than the background rate.

*Prostate cancer* risk is also higher in BRCA2 compared to BRCA1 carriers. Cumulative prostate cancer risk assessed in 173 BCLC-BRCA2 families was 7.5% (95% CI 5.7–9.3%) by age 70 years, corresponding to a RR of 4.65 (95% CI 3.48–6.22), which may include a detection bias. A Dutch study of BRCA2 carriers reached similar conclusions, with a cumulative risk of 5.2% (95% CI 1.7–8.7%) by age 70 years, reflecting an RR of 2.5 (95% CI 1.6–3.8) (van Asperen *et al*, 2005). BCLC family-based studies found that prostate cancer risk in BRCA2 carriers depend on age and BRCA2 mutation location. Relative prostate cancer risk was higher in men younger than 65 years (RR=7.3 (95% CI 4.7–11.5) than in older men (RR 3.4 (85% CI 2.3–4.9) (The BCLC, 1999), and mutations outside the OCCR were associated with higher risks (33.6% (95% CI 25.1–44.1%) by age 80 years) than mutations within the OCCR (19.2% (95% CI 10.7–33.1%) by age 80 years) (Thompson and Easton, 2001). The lower risk in OCCR mutations (OR=0.52, 95% CI 0.24–1.00) may explain why prostate cancer risk has not been consistently elevated in studies of BRCA2 in AJ (Liede *et al*, 2004). It is less clear whether BRCA1 mutations increase prostate cancer risk. In the BCLC-BRCA1 families, there was some evidence of an increased risk of prostate cancer for men younger than 65 years (RR 1.82, 95% CI 1.01–3.29), but not for those aged 65 years or older (RR=0.84, 95% CI 0.53–1.33) (Thompson and Easton, 2002). Studies in AJ have yielded conflicting results, but in general BRCA1 mutations have a limited contribution to prostate cancer risk in this population (Liede *et al*, 2004).

## RISK FOR OTHER CANCERS

In addition to breast and ovarian cancer in women, and breast and prostate cancer in men, BRCA1 and BRCA2 carriers may be at higher risk for additional malignancies. However, the absolute risks for specific cancers at other sites are small (Gruber and Petersen, 2002; Thompson and Easton, 2002). Risk for malignancies at other sites has so far been addressed using two designs: family-based studies, which compare the observed *vs* expected number of cases of a specific malignancy, and studies which compare rates of BRCA1/BRCA2 mutations in unselected cases of a specific malignancy with mutation rates in controls or in the general population. In family-based studies, diagnoses other than breast or ovarian cancer were confirmed by pathology or clinical records in only about half of the cases (The BCLC, 1999, Thompson and Easton, 2002; van Asperen *et al*, 2005), whereas in studies of mutation rates in specific cancers, all cases are confirmed by pathology records. This is an important issue when risk estimates are based on a small number of specific cancer cases. Thus, reports of possible excess risk of other gynaecological cancers (e.g. cervix and uterus), and gall bladder and bile duct cancer may represent misclassifications of ovarian and pancreatic cancer, respectively, and liver and bone cancers may represent distant metastases from other sites.

Colon and rectal cancer risk was originally thought to be higher in BRCA1, but not BRCA2 carriers (The BCLC, 1999; Thompson and Easton, 2002). However, in the largest family-based study, combined colorectal cancer risk was not increased (Gruber and Petersen, 2002) and a large study of 225 unselected AJ colon cancer cases did not find increased rates of BRCA1 or BRCA2 mutations (Niell *et al*, 2004). Malignant melanoma, both cutaneous and ocular, has been reported in BRCA2 families, and an excess risk has been reported in the BRCA2-BCLC families (The BCLC, 1999). This has not been confirmed in a smaller Dutch study (van Asperen *et al*, 2005), and studies of unselected uveal melanoma cases have not shown excess rates of BRCA2 mutations (Hearle *et al*, 2003). Taken together, this suggests that melanoma risk may be elevated in specific BRCA2 families, but not for all BRCA2 carriers.

Pancreatic cancer is currently the only additional malignancy for which there is unequivocal evidence for increased risk in BRCA1 and BRCA2 carriers, although the absolute risk is small. In BRCA1 carriers pancreatic cancer risk carriers by age 70 years has been estimated to be 1.16% (95% CI 0.83–1.61%) in men, and 1.26% (95% CI 0.92–1.72%) in women, reflecting a two-fold RR (Thompson and Easton, 2002). In BRCA2 carriers, the largest study is that of the BCLC which included data on 566 malignancies other than breast or ovarian cancers. This study estimated pancreatic cancer risk in BRCA2 carriers by age 70 years to be 2.1% (95% CI 1.2–3.0%) in men, and 1.5% (95% CI 0.9–2.1%) in women, reflecting a combined RR of 3.51 (95% CI 1.87–6.58) (The BCLC, 1999). A smaller Dutch study, based on 199 malignancies other than breast or ovarian cancer, estimated pancreatic cancer risk in BRCA2 carriers by age 70 years at 4.1% (95% CI 1.0–7.3%) in men and 1.4% (95% CI 0–3.4%) in women (Van Asperen *et al*, 2005). These risk estimates are also consistent with the 4–7% rate of BRCA1 and BRCA2 mutations among unselected pancreatic cases (reviewed in Liede *et al*, 2004).

## CONCLUSION

Breast and ovarian cancer risk in BRCA1 and BRCA2 carriers is a complex trait, affected by genetic modifiers and nongenetic factors. While further studies are necessary to define and quantify risk-factor effects, even now the best approach for the individual patient is perhaps a personalised one, which takes into account family history, ascertainment, and known environmental risk factors.
